# Exposed facet-controlled N_2_ electroreduction on distinct Pt_3_Fe nanostructures of nanocubes, nanorods and nanowires

**DOI:** 10.1093/nsr/nwaa088

**Published:** 2020-04-30

**Authors:** Wu Tong, Bolong Huang, Pengtang Wang, Qi Shao, Xiaoqing Huang

**Affiliations:** College of Chemistry, Chemical Engineering and Materials Science, Soochow University, Suzhou 215123, China; Department of Applied Biology and Chemical Technology, Hong Kong Polytechnic University, Hong Kong, China; College of Chemistry, Chemical Engineering and Materials Science, Soochow University, Suzhou 215123, China; College of Chemistry, Chemical Engineering and Materials Science, Soochow University, Suzhou 215123, China; College of Chemistry, Chemical Engineering and Materials Science, Soochow University, Suzhou 215123, China

**Keywords:** Pt_3_Fe, nanowire, high-index, facet-controlled, N_2_ reduction

## Abstract

Understanding the correlation between exposed surfaces and performances of controlled nanocatalysts can aid effective strategies to enhance electrocatalysis, but this is as yet unexplored for the nitrogen reduction reaction (NRR). Here, we first report controlled synthesis of well-defined Pt_3_Fe nanocrystals with tunable morphologies (nanocube, nanorod and nanowire) as ideal model electrocatalysts for investigating the NRR on different exposed facets. The detailed electrocatalytic studies reveal that the Pt_3_Fe nanocrystals exhibit shape-dependent NRR electrocatalysis. The optimized Pt_3_Fe nanowires bounded with high-index facets exhibit excellent selectivity (no N_2_H_4_ is detected), high activity with NH_3_ yield of 18.3 μg h^−1^ mg^−1^_cat_ (0.52 μg h^−1^ cm^−2^_ECSA_; ECSA: electrochemical active surface area) and Faraday efficiency of 7.3% at −0.05 V versus reversible hydrogen electrode, outperforming the {200} facet-enclosed Pt_3_Fe nanocubes and {111} facet-enclosed Pt_3_Fe nanorods. They also show good stability with negligible activity change after five cycles. Density functional theory calculations reveal that, with high-indexed facet engineering, the Fe-3d band is an efficient *d-d* coupling correlation center for boosting the Pt 5d-electronic exchange and transfer activities towards the NRR.

## Teaser

We have reported facile synthesis of Pt_3_Fe nanocrystals with tunable morphologies (nanocubes, nanorods and nanowires) to evaluate the performance of N_2_ electroreduction on different exposed surface structures.

## INTRODUCTION

Ammonia (NH_3_), an essential composition for fertilizer feedstock, industrial and chemical precursors [1], not only plays a vital role in the development of ammonia fuel cells but is also a promising candidate for the hydrogen economy because of its high energy density and large hydrogen capacity [2]. However, from a thermodynamic point of view, the high bond energy of the triple bond in nitrogen (N_2_) makes it an intricate multi-step reaction to convert N_2_ to NH_3_ [3]. Currently, the Haber-Bosch method is most widely applied because efficient synthesis of NH_3_ from N_2_ and H_2_ has reached industrial-scale yields [4]. However, this method requires conditions of high temperature of 400–500 °C and pressure of 200–250 bar [5]. It also consumes 1–2% of the global annual energy supply and is responsible for >1% of global CO_2_ emissions [6]. Therefore, a more effective process for NH_3_ synthesis under mild conditions is highly desirable.

Recently, tremendous efforts for converting N_2_ to NH_3_ have been made in pursuit of efficient and sustainable catalysis with use of biocatalytic, photocatalytic and electrocatalytic methods [7–14]. One possible strategy for NH_3_ synthesis is electroreduction of N_2_ to NH_3_ in which the N_2_ reduction reaction (NRR) process can be operated by renewable electricity energy, and operated at mild temperature and pressure [15–18]. There have been several investigations on NRR with noble-metal catalysts (Ru [19], Au [20], Pd [21] and Rh [22]), as summarized in Table S1. More recently, several efforts have been devoted to optimizing electrocatalysts to enhance NRR, including size regulation [23], crystal engineering [24], ion incorporation [25], introduction of defect sites [26,27] and component regulation [28]. However, to the best of our knowledge, surface structure regulation, as one of the most effective strategies to precisely tune catalytic properties, has not yet been demonstrated for NRR.

Previous research revealed that the NRR performance is gravely limited by linear scaling of the two vital intermediate energetics between *N_2_H and *NH_2_ (* indicates the adsorption site). Further theoretical analyses disclose that Fe and Pt atoms can effectively address the energetics of *N_2_H and *NH_2_, respectively, synergistically providing efficient active sites to enhance NRR activity [29,30]. Herein, we report a facile method for selectively growing Pt_3_Fe nanocubes (NCs), Pt_3_Fe nanorods (NRs) and Pt_3_Fe nanowires (NWs) (Supplementary Fig. 1), which give rise to active and stable electrocatalysts for NRR. Optimized Pt_3_Fe NWs bounded with high-index facets exhibit much enhanced NH_3_ yield (18.3 μg h^−1^ mg^−1^_cat_, 0.52 μg h^−1^ cm^−2^_ECSA_; ECSA: electrochemical active surface area), Faradaic efficiency (7.3%) and selectivity (no N_2_H_4_ is detected) at −0.05 V versus reversible hydrogen electrode (RHE), and are much better than those of {200} facet-enclosed Pt_3_Fe NCs and {111} facet-enclosed Pt_3_Fe NRs. The Pt_3_Fe NWs also show durable stability with negligible activity decay for five cycles. Density functional theory (DFT) calculation reveals that, on the high-indexed surface engineering, strong orbital interaction between Pt and neighboring Fe sites induces an obvious correlation effect for boosting up Pt-5d electronic activities for efficient NRR.

## RESULTS AND DISCUSSION

**Figure 1.**
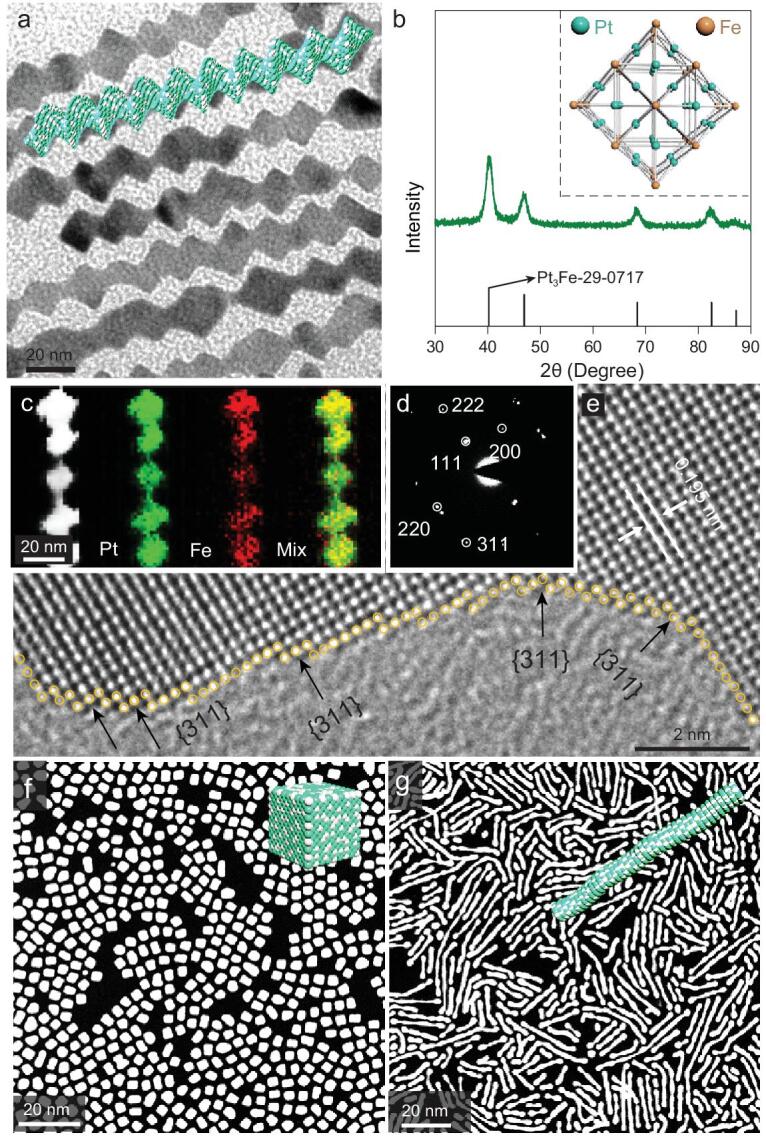
(a) TEM image and schematic illustration, (b) XRD pattern and crystal structure, (c) elemental mappings and HAADF-STEM image, (d) SAED image (white circles indicate the diffraction spots) and (e) spherical aberration correction HRTEM image of Pt_3_Fe NWs. HAADF-STEM images and schematic illustrations of (f) Pt_3_Fe NCs and (g) Pt_3_Fe NRs.

A simple wet-chemical method is adopted to control the synthesis of Pt_3_Fe nanocrystals. Taking Pt_3_Fe NWs synthesis as an example, potassium tetrachloroplatinate (II) (K_2_PtCl_4_) and iron nonacarbonyl (Fe_2_(CO)_9_) are chosen as metal precursors, ribose is used as the reducing agent, cetyltrimethyl ammonium chloride (CTAC) and oleylamine (OAm) are applied as the surfactant and solvent, respectively. Uniform Pt_3_Fe NWs with average diameter of 15 nm and zigzag border along the whole length are obtained (Fig. 1a and Supplementary Fig. 2), characterized by high-angle annular dark-field scanning transmission electron microscopy (HAADF-STEM) and TEM. The X-ray diffraction (XRD) pattern (Fig. 1b) of Pt_3_Fe NWs shows distinct diffraction peaks at 40.32, 46.92, 68.42, 82.52 and 87.2^o^, which are readily indexed to (111), (200), (220), (311) and (222) reflections of face-centered cubic (*fcc*) Pt_3_Fe (JCPDS number 29–0717), being consistent with those of Pt_3_Fe NCs and Pt_3_Fe NRs (Supplementary Fig. 3) [31]. The crystal structure model of the Pt_3_Fe NWs represents a primitive cubic structure. It is composed of a periodic square matrix of Fe and Pt, which are located at the corner and face center of each unit cell, respectively. The HAADF-STEM image and elemental mappings show the elemental distributions of Pt and Fe (Fig. 1c). The Pt (green), Fe (red) and mixed images indicate that all elements are evenly distributed on the Pt_3_Fe NWs, confirming the alloyed structure (Supplementary Fig. 4). The selected area electron diffraction (SAED) image (Fig. 1d) further indicates that the Pt_3_Fe NWs have good crystallinity and *fcc* structure. Spherical aberration correction HRTEM images reveal the distinct lattice fringes. The measured value of lattice spacing is 0.195 nm. In addition, a high-index facet of {311} can be readily observed (Fig. 1e and Supplementary Fig. 5), which is reported to exhibit much higher catalytic performance compared to most common facets ({200} and {111}) because of the high density of atomic steps and ledges [32–34]. Notably, when ribose is replaced by maltose, while maintaining the other synthetic parameters unchanged, Pt_3_Fe NCs with average diameter of 5 nm are obtained (Fig. 1f and Supplementary Fig. 6). Meanwhile, Pt_3_Fe NRs with average diameter of 3 nm are realized by merely changing the precursor of Pt (Fig. 1g; Supplementary Figs 7 and 8). Detailed X-ray photoelectron spectroscopy (XPS) is carried out to determine the electronic properties of surface Pt atoms. Compared with commercial Pt/C, the Pt^0^ 4f_7/2_ binding energy of Pt_3_Fe nanocrystals has a negative shift from 71.2  to 70.6 eV, indicating that the charge is transferred from Fe to Pt, because of the lower electronegativity of Fe (Supplementary Fig. 9a) [35]. We can see that the Pt 4f spectra show two distinct peaks, which are assigned to the Pt 4f_7/2_ and Pt 4f_5/2_ orbit levels. Each peak can be further split into two doublets, which are assigned to Pt^0^ and Pt^2+^ chemical valence states. It is clearly shown that the majority of the Pt of Pt_3_Fe is in mainly metallic state. The Pt(II)/Pt(0) ratio is summarized in Table S2. The XPS spectra of Fe 2p reveal two distinct peaks at 710.3  and 724.1 eV, which are assigned to the characteristic peaks of Fe 2p_3/2_ and Fe 2p_1/2_ orbit levels, respectively. The results indicate that the majority of the Fe is mainly in an oxidized state (Supplementary Fig. 9b) [36]. The lattice spacings of Pt_3_Fe NCs and Pt_3_Fe NRs are measured to be 0.195  and 0224 nm, corresponding to the {200} and {111} facets, respectively (Supplementary Figs 10 and 11). Hence, well-defined Pt_3_Fe nanocrystals bounded with distinct facets have been successfully created and can be adopted as ideal model electrocatalysts for fundamental understanding of the relationship between surface structure and catalysis.

**Figure 2.**
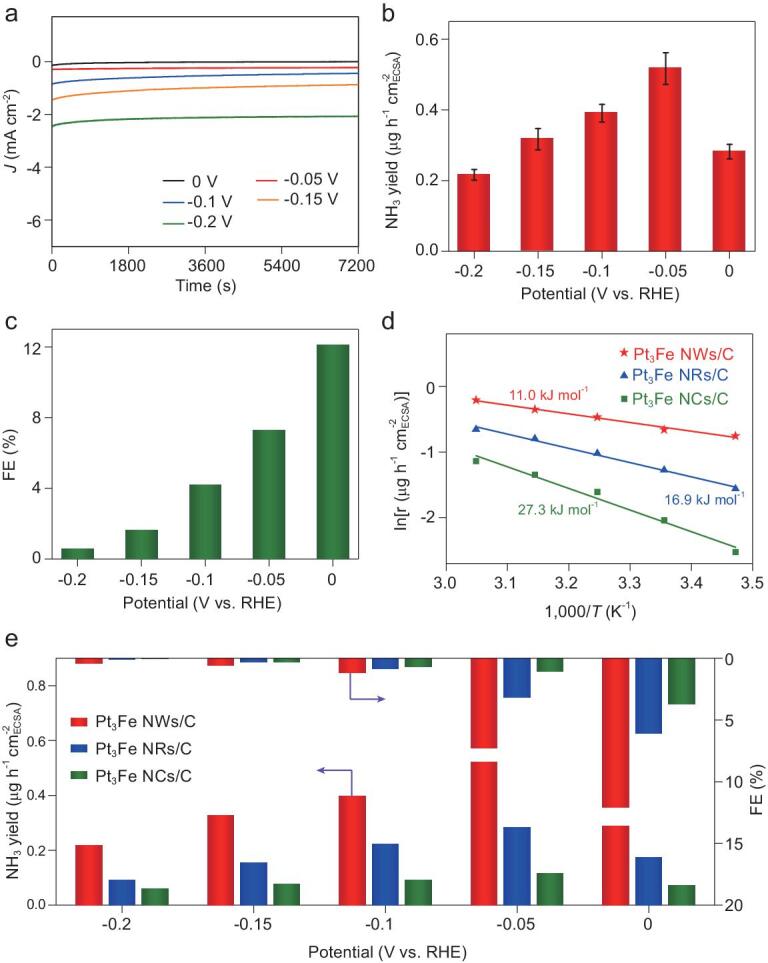
(a) I-t curves, (b) NH_3_ yield and (c) FE of Pt_3_Fe NWs/C at different applied potentials. (d) Apparent activation energy for NRR with different Pt_3_Fe electrocatalysts. (e) Histograms of the NH_3_ yield and FE of Pt_3_Fe NCs/C, Pt_3_Fe NRs/C and Pt_3_Fe NWs/C. The error bars in (b) indicate the standard deviations of three independent tests in the same conditions.

Pt NCs, Fe_3_O_4_ NPs (Supplementary Fig. 12) and Pt_3_Fe nanocrystals were first loaded onto carbon (C, Vulcan XC-72) to investigate the NRR properties. The resulting electrocatalysts were called Pt NCs/C, Fe_3_O_4_ NPs/C, Pt_3_Fe NCs/C, Pt_3_Fe NRs/C and Pt_3_Fe NWs/C. A schematic for the electrochemical NRR is shown in Supplementary Fig. 13. During each NRR measurement, pure N_2_ flowed into the cathode electrolyte at a flow rate of 30 standard mL/min, wherein N_2_ combined with electrons to form N_2_ reduction product. Using Pt_3_Fe nanocrystals as the cathodic catalysts, only NH_3_ without the by-product of N_2_H_4_ could be detected, highlighting the good selectivity for NH_3_ (Supplementary Fig. 14). The standard calibration curves are given in Supplementary Figs 15 and 16. Saturated calomel reference electrode is calibrated on reversible hydrogen electrode (Supplementary Fig. 17). During the NRR progress, the Pt_3_Fe NWs/C exhibited negligible decay in current density under different applied potential, indicating the good corrosion resistance ability (Fig. 2a). The Pt NCs/C (Supplementary Fig. 18) had very poor NRR activity compared with Pt_3_Fe NCs/C, indicating that introduction of Fe is essential for the NRR. In addition, the NRR activity of Pt_3_Fe NRs/C exhibited much improvement compared with Pt_3_Fe NCs/C. The average NH_3_ yield and Faraday efficiency (FE) of the Pt_3_Fe NWs/C under different applied potentials is given in Fig. 2b and c. The NRR was performed at 0 V, the measured yield of NH_3_ was 10.2 μg h^−1^ mg^−1^_cat_ (0.29 μg h^−1^ cm^−2^_ECSA_) with the highest FE of 12.3%. The NH_3_ yield increased to a maximum value of 18.3 μg h^−1^ mg^−1^_cat_ (0.52 μg h^−1^ cm^−2^_ECSA_) until −0.05 V, indicating that it consumed less energy to effectively convert N_2_ into NH_3_. The NRR performance of Pt_3_Fe NWs/C was much better than that of the Fe_3_O_4_ catalysts (Supplementary Fig. 19). The maximum yield of NH_3_ was further quantitatively determined by an indophenol blue method [37]. As observed, the calculation value of NH_3_ yield was close to the quantitative result from the Nessler reagent method (Supplementary Figs 20 and 21). The FE linearly decreased when it reached a more negative potential, because of the existence of a competition reaction between NRR and hydrogen evolution reaction (HER) [38]. We compared the NRR activity with different reaction temperatures at −0.05 V to assess the apparent activation energy and investigate the different exposed facets effect of Pt_3_Fe nanocrystals. As observed, the NH_3_ yield enhanced with increasing reaction temperature due to faster mass transfer rate of reactants (Supplementary Fig. 22). The estimated apparent activation energies were 11.0, 16.9 and 27.3 kJ mol^−1^ for Pt_3_Fe NWs/C, Pt_3_Fe NRs/C and Pt_3_Fe NCs/C, respectively (Fig. 2d). That is, the Pt_3_Fe NWs/C bounded with high-index facets could significantly decrease the apparent activation energy and hence enhance the NRR activity. Significantly, the NH_3_ yield and FE of Pt_3_Fe NWs/C were higher than those of Pt_3_Fe NCs/C and Pt_3_Fe NRs/C (Fig. 2e), indicating that different surface structures of Pt_3_Fe nanocrystals indeed have essential influence on the NRR activity and the high-index facets of Pt_3_Fe nanocrystals play a vital role in the improvement of NRR activity.

We then carefully examined the N source of the produced NH_3_. There were no distinguishable peaks in the N 1s region, indicating that no nitrogenous species existed on the surface of synthetic catalysts (Supplementary Fig. 23). Replacing N_2_ by Ar, while maintaining other experimental parameters unchanged, no NH_3_ was detected (Supplementary Fig. 24). The same result was observed when Pt_3_Fe NWs/C was replaced by carbon powder loaded on carbon paper (Supplementary Fig. 25). The amount of NH_3_ increased linearly to the electrocatalysis time in N_2_-saturated electrolyte, which indicated that the produced NH_3_ came from the NRR process (Supplementary Fig. 26). Considering the small amounts of NH_3_ and NO_x_ in atmosphere and feeding gas, we kept high-purity Ar and N_2_ continuously flowing into the KOH electrolyte without applied potential. The UV-vis results (Supplementary Figs 27 and 29) revealed that no NH_3_ and NO_x_ were detected. Finally, ^15^N (99% ^15^N atom) isotope labeling experiments were carried out to further confirm the NH_3_ source. After continuous electrolysis at −0.05 V using ^15^N_2_ as the supplying gas, the ^1^H nuclear magnetic resonance spectra show a double coupling peak of ^15^NH_4_^+^ without the triple coupling peak of ^14^NH_4_^+^. Hence, the result confirmed that the NH_3_ was derived from the electroreduction of N_2_ in the presence of Pt_3_Fe NWs/C (Supplementary Fig. 30). In addition, by varying the N_2_ flow rate, the current density exhibited small change. The tiny fluctuation of FE and NH_3_ yield implied that N_2_ diffusion was not the rate-determining step (Supplementary Fig. 31) [39].

The intrinsic reason for higher catalytic activity of Pt_3_Fe NWs/C was investigated. The Tafel slope is a vital parameter to evaluate the HER mechanism [40,41]. The higher value of 163.8 mV dec^−1^ gained from Pt_3_Fe NWs/C suggested its sluggish HER kinetics, which may in turn enhance the NRR performance (Supplementary Fig. 32), further confirmed by theoretical calculation (Supplementary Fig. 33). After that, surface valance spectra was created to investigate the relationship between electronic effect and the binding strength of adsorbates. As observed, the d-band center shifts upwards from Pt NCs/C (−3.69) to Pt_3_Fe NCs/C (−3.31) when introducing Fe to Pt NCs/C. For transition metals, the lower binding energy of d-band center would cause weaker bonding between the adsorbates and metal surface [42–44]. Accordingly, the Pt_3_Fe NWs/C bounded with high-index facets could enable strong bonding with N_2_ and may boost NRR activity (Supplementary Fig. 34).

**Figure 3.**
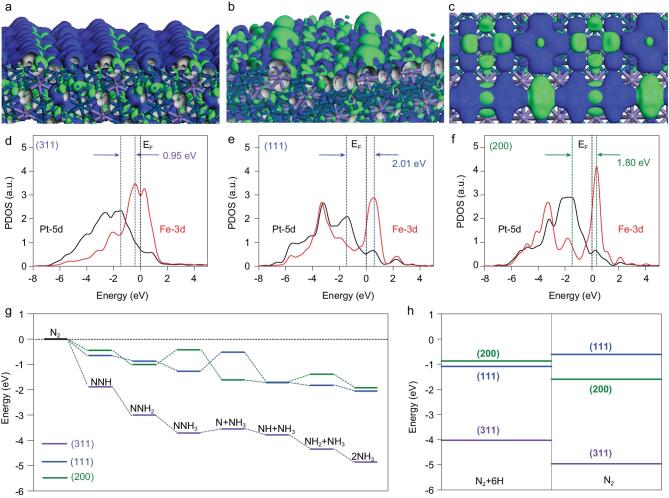
(a–c) The real spatial contour plots of bonding and anti-bonding orbitals near E_F_ on Pt_3_Fe (311), (111) and (200) surfaces, respectively. (d–f) PDOSs of d-bands for surface Pt-5d and Fe-3d sites on the (311), (111) and (200) are given, respectively. (g) The NRR energetic pathway on the surfaces of (311), (111) and (200), respectively. (h) Direct adsorption comparison for N_2_+6H and N_2_ on the surfaces of (311), (111) and (200), respectively.

We used DFT calculations to further interpret the NRR activity differences of Pt_3_Fe (311), (111) and (200). The bonding and antibonding orbitals near the Fermi level (E_F_) exhibit an electron-rich distribution on the Pt_3_Fe (311) surface (Fig. 3a), while (111) and (200) surfaces present less electron-localizing on the surface (Fig. 3b and c). The preeminent d-electron exchange and transfer activities (d-EXTA) on the high indexed surface have been demonstrated through projected partial density of states (PDOSs) analysis (Fig. 3d–f). The edge of dominant peak of Pt-5d band reflects a direct determination of 5d-EXTA for efficient N_2_ fixation. Taking this trend, on the (311) surface, the Fe-3d orbital clearly merges at the E_F_ without an evident gap between the *e_g_* and *t_2g_* components. The energetic interval between Pt-5d and Fe-3d is nearly 0.95 eV, which cost less energetic barrier for transferring d-electrons above E_F_ to N_2_ 2p orbital (Fig. 3d). The (111) and (200) present nearly two-times higher to freely cross E_F_ (Fig. 3e and f). The comparison of energetic trends reveals that the NRR pathway on the (311) is the most energetically beneficial with an energetic barrier of merely 0.16 eV and overall gain of −4.86 eV. However, the (111) and (200) denote a higher energy gain of about −2.0 eV and the energy barriers are 0.75  and 0.34 eV, respectively (Fig. 3g). In the distal reaction pathway, the N atom furthest away from the catalyst surface preferentially undergoes hydrogenation, to form the first NH_3_ molecule. The NH_3_ molecule is released after the N≇N triple bond breaks. The remaining N atom continues under hydrogenation to generate another NH_3_ molecule. According to the above results, the reaction path of our work could be a distal reaction pathway. We further reason that the better activity of NRR on the (311) is attributed to the excellent energetic preference of N_2_ fixation, while the underperformance of (200) for NRR is ascribed to overbinding of adsorbing H for efficient N-hydrogenation (Fig. 3h). The energetic trend is consistent with analysis of electronic activities from both experimental and theoretical perspectives.

**Figure 4.**
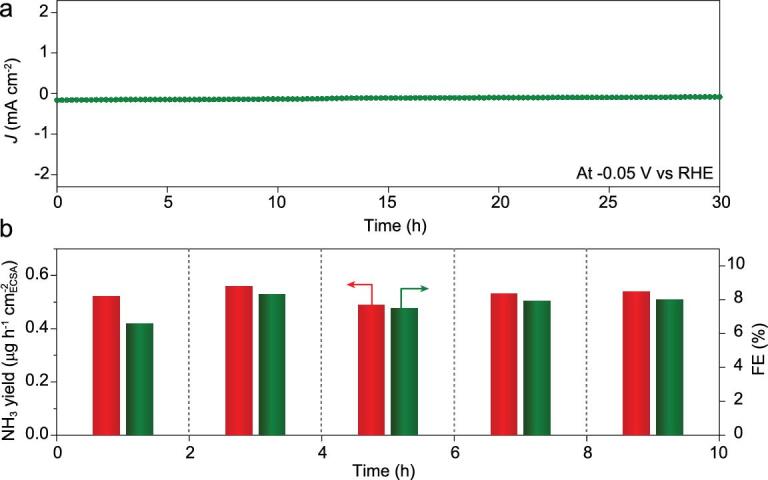
(a) I-t curve of the Pt_3_Fe NWs/C in N_2_-saturated KOH electrolyte at −0.05 V and (b) NH_3_ yield and FE calculated after each cycle at −0.05 V.

A chronoamperometric test was first conducted at −0.05 V in N_2_-saturated KOH electrolyte to evaluate the durability of Pt_3_Fe NWs/C. The current density (Fig. 4a) exhibited negligible decay after 30 h electrolysis. The stability of Pt_3_Fe NWs/C was also evaluated by successive cycle electrolysis at −0.05 V. After five successive cycles, the total current density exhibited no evident fluctuation (Supplementary Fig. 35). The NH_3_ yield and FE of Pt_3_Fe NWs/C were measured after each cycle, with no obvious changes (Fig. 4b). After stability testing, TEM image, elemental mapping (Supplementary Fig. 36) and SEM-EDS (Supplementary Fig. 37) of the Pt_3_Fe NWs/C confirmed that its structure and composition were largely maintained, demonstrating that the Pt_3_Fe NWs/C was stable enough for NRR.

## CONCLUSION

In summary, we have demonstrated facile synthesis of Pt_3_Fe nanocrystals with tunable morphologies (NC, NR and NW) to evaluate the NRR performance on different exposed facets. The detailed studies show that the Pt_3_Fe nanocrystals exhibit shape-dependent electrocatalytic activity towards NRR. Notably, the Pt_3_Fe NWs bounded with high-index facets exhibit much improvement in NH_3_ yield (18.3 μg h^−1^ mg^−1^_cat_, 0.52 μg h^−1^ cm^−2^_ECSA_), FE (7.3%) as well as selectivity (no N_2_H_4_ is detected) at −0.05 V under ambient conditions, which are much better than that those of {200} facet-enclosed Pt_3_Fe NCs and {111} facet-enclosed Pt_3_Fe NRs. The Pt_3_Fe NWs also show durable electrochemical stability with no obvious activity decay after five successive electrolysis cycles. DFT calculation reveals that strong d-d coupling between Pt and Fe sites bridges the electron transfer for prominent NRR. This work provides the first example of the fundamental correlation between exposed surfaces and NRR performances of distinct nanocrystals.

## METHODS

### Preparation of Pt_3_Fe NWs

In preparation of monodisperse Pt_3_Fe NWs, K_2_PtCl_4_ (10.4 mg), Fe_2_(CO)_9_ (4.6 mg), ribose (45 mg), CTAC (32 mg) and OAm (5 mL) were added to a reaction bottle (volume: 35 mL), which was capped and ultrasonicated for 1 h. The reaction bottle was heated from room temperature to 180°C within 0.5 h and maintained at 180°C for 5 h in an oil bath. After cooling to room temperature, the obtained products were collected by centrifugation and washed three times with a cyclohexane/ethanol mixture.

### Preparation of Pt_3_Fe NRs

The synthesis of monodisperse Pt_3_Fe NRs was similar to that of Pt_3_Fe NWs, except Pt(acac)_2_ (9.8 mg) was used as precursor and the amount of ribose was 90 mg at the beginning.

### Preparation of Pt_3_Fe NCs

The synthesis of monodisperse Pt_3_Fe NCs was similar to that of Pt_3_Fe NWs, except maltose (108 mg) was used as reductant at the beginning.

### Preparation of Pt NCs

In preparation of monodisperse Pt NCs, Pt(acac)_2_ (10 mg), PVP (200 mg), formaldehyde solution (40%, 2.5 mL) and benzyl alcohol (10 mL) were added to a reaction bottle. After sonication for 0.5 h, the homogeneous solution was transferred to a 20 mL Teflon-lined stainless autoclave and then heated at 150°C for 10 h. The product was collected via centrifugation and further washed with an ethanol-acetone mixture.

### Preparation of Fe_3_O_4_ NPs

In preparation of monodisperse Fe_3_O_4_ NPs, Fe_2_(CO)_9_ (9.2 mg), ribose (45 mg), OAm (4.8 mL) and OAC (0.2 mL) were added to a reaction bottle, which was capped and then ultrasonicated for 1 h. The reaction bottle was heated from room temperature to 200°C within 0.5 h and maintained at 200°C for 5 h in an oil bath. After cooling to room temperature, the obtained products were collected by centrifugation and washed three times with a cyclohexane/ethanol mixture.

### Catalytic measurements

Firstly, different morphology of Pt_3_Fe nanocrystals, Fe_3_O_4_ NPs, commercial carbon, cyclohexane (1 mL) and ethanol (8 mL) were added to a reaction bottle. The Pt NCs, commercial carbon, ethanol (1 mL) and acetone (8 mL) were also added to a reaction bottle. After sonication for 1 h, the homogeneous solution was centrifuged and dried naturally to obtain a powder. Then, the powder was annealed at 150°C for 1 h under ambient atmosphere. To prepare catalysts, the above powder (5 mg), Nafion solution (6 μL, 5 wt%) and absolute isopropyl alcohol (500 μL) were mixed and sonicated for 0.5 h to form homogeneous ink, which was then dropped (10 μL) evenly on carbon paper with geometric area of 1 × 1 cm^2^. The carbon paper was dried under ambient conditions.

The electrochemical tests were performed in a gas-tight two-chamber electrolytic cell separated by Nafion 115 membrane. Before electrochemical NRR measurements, the Nafion 115 membrane was first pretreated in 80°C H_2_O_2_ (5%) aqueous solution for 1 h and then washed in 80°C ultrapure water for another 1 h. The electrochemical experiments were conducted on a CHI660E electrochemical analyzer (CHI Instruments) using a three-electrode configuration (working electrode of synthetic catalysts, reference electrode of saturated calomel electrode and counter electrode of carbon rod). The mentioned potentials have been converted to RHE. The cyclic voltammetry (CV) tests were carried out in 0.1 M HClO_4_ solution with a scan rate of 50 mV s^−1^ under ambient conditions. The ECSA was obtained by integrating the hydrogen adsorption charge (*Q_H_*) between −0.25 V and 0.1 V on the CV curves. The value of adsorbed single-layer hydrogen (*q_H_*) on the Pt surface is 210 μC cm^−2^ and the formula is *ECSA* = *Q_H_/*(*q_H_ × m*) (Supplementary Fig. 38).

Potentiostatic tests were carried out in electrochemical NRR. Before the measurement, highly pure N_2_ gas was flowed continuously into the cathode electrolytic cell with a proper position for 0.5 h.

### Calculation of NH_3_ yield, Faraday efficiency and apparent energy

In NRR tests, FE is defined as the amount of charge for the synthesis of NH_3_ divided by the total charge through the electrodes during the electrolysis process. The NH_3_ yield was determined by a colorimetric method using Nessler's reagent. The formation of each NH_3_ molecule requires three electrons, hence the FE of NH_3_ can be determined by the below formula:

$$\begin{equation*}
\ln\!\! K = \frac{{ - {E_a}}}{{RT}} + C.
\end{equation*}$$

The NH_3_ yield was determined by the below formula:

$$\begin{equation*}
{F E} = \frac{3 \times F \times C_{\rm NH_{3}} \times {\rm V}}{17 \times {\rm Q}},
\end{equation*}$$

where $Q$: quantity of electric charge; $F{:}$ Faraday constant, 96485 C mol^−1^; $V {:}$ the KOH electrolyte volume; $C_{\rm NH_3}{:}$ the calculated NH_3_ concentration; *t:* the reduction time; $A$: the ECSA of the catalysts.

According to the Arrhenius equation, the apparent energy calculations process is below:

$$\begin{equation*}V_{{\rm NH_3}}=\frac{C_{\rm NH_{3}} \times {\rm V}}{{\rm t \times A}},\end{equation*}$$

where *K*: NH_3_ yield rate at temperature T; *R*: Molar gas constant (J mol^−1^ K^−1^); *E_a_*: Apparent energy (kJ mol^−1^), *T*: Absolute reaction temperature (K). We plot lnK against 1000/T, and the slope is k, so E_a_ = -kR.

### Determination of ammonia yield

The yield of NH_3_ was determined via a colorimetric method using Nessler's reagent. The calibration curve was obtained as follows: first, a series of reference solutions was prepared, by pipetting known NH_4_Cl solutions and 0.1 M KOH working electrolyte into colorimetric tubes. These were made up to the mark (10 mL) with 0.1 M KOH solution. Next, 1 mL of 0.2 M potassium sodium tartrate (KNaC_4_H_4_O_6_, chelating soluble metal ion) ultrapure water solution was pipetted into each of the tubes and these were mixed thoroughly, followed by pipetting of 1 mL Nessler's reagent into each of the tubes and further mixing. The mixed solutions were left for 0.5 h. Using a blank solution for background correction, the absorbance of the solutions was measured at 425 nm in a 10 mm glass cuvette. The calibration curve (y = 0.162x-0.005, R^2^ = 0.998) exhibited a good linear relationship between absorbance and NH_4_^+^ concentration according to three independent calibration tests.

### Determination of hydrazine hydrate

The hydrazine hydrate was determined by a colorimetric method using Watt-Chrisp reagent. Para (dimethylamino) benzaldehyde (5.99 g), concentrated HCl (30 mL) and absolute ethanol (300 mL) were mixed as color reagents. A calibration curve was obtained as follows: first, different reference solutions were prepared, by pipetting hydrazine hydrate-nitrogen 0.1 M HCl solution into colorimetric tubes. These were made up to 5 mL with diluted hydrochloric acid electrolyte (pH = 1), then 5 mL was pipetted above the color reagent and this was left to stand for 0.5 h for color development under ambient conditions. The absorbance of the color solution was measured at 455 nm with a 10 mm glass cuvette, and the yields of hydrazine were determined by the standard calibration curve using a mixture of 5 mL remaining solution and 5 mL color reagent. The calibration curve (y = 1.208x-0.088, R^2^ = 0.977) was obtained with hydrazine monohydrate solutions of different known concentrations, and exhibited a good linear relationship between absorbance and N_2_H_4_·H_2_O concentration according to three independent calibration tests.

### Determination of NO_x_

The concentration of NO_x_ was measured with a colorimetric method using N-(-1-naphthyl) ethylenediamine dihydrochloride as color reagent. A mixture of 0.5 g sulfanilic acid, 5 mg n-(1-naphthyl)-ethylenediamine dihydrochloride, 90 mL H_2_O and 5 mL acetic acid was stirred to form a homogeneous solution. This was transferred to a 100 mL volumetric flask to obtain the color agent. A mixture of 1 mL electrolyte and 4 mL color agent was left for 0.5 h in the dark. The absorbance of the solutions was measured at 540 nm from the UV-vis absorption spectrum. The calibration curve was obtained by using different known concentrations of potassium nitrite solution in 0.1 M KOH.

### 15 ^15^N isotope labeling experiment

An ^15^N isotopic labeling experiment was carried out to verify the source of produced ammonia. After ^15^N_2_ electroreduction at −0.05 V in KOH electrolyte for 10 h, the obtained product was qualitatively determined by ^1^H nuclear magnetic resonance (NMR, Agilent 600 MHz). In detail, 30 mL of the electrolytic solution was moved out and then acidized to pH ∼ 3. The solution was concentrated to 2 mL at 80°C. Afterwards, 0.9 mL of concentrated solution and 0.1 mL D_2_O containing 100 ppm dimethyl sulphoxide (99.99%) as an internal standard were mixed for ^1^H NMR test.

### DFT calculations

The DFT+U calculations were carried out with CASTEP code [45]. In this framework, we use rotationally invariant (Anisimov type) functional DFT+U [46]. The Hubbard U parameter is self-consistently determined for the pseudized C-2p, Fe-3d and Pt-5d orbital by the new linear response way [47–53]. The geometry optimization used the Broyden-Fletcher-Goldfarb-Shannon (BFGS) algorithm in all DFT+U calculations. The PBE functional was chosen for PBE+U calculations with a kinetic cutoff energy of 750 eV, with the valence electron states expressed in a plane-wave basis set. The ensemble DFT (EDFT) method of Marzari *et al*. [54] was used for convergence on the transition metal contained compounds.

The 2 × 2 × 1 supercell of Pt_3_Fe (311) surface model was chosen with 112 atoms (i.e. Pt_84_Fe_28_) and seven layers thick. The 2 × 2 × 1 supercell of Pt_3_Fe (111) surface model was chosen with 128 atoms (i.e. Pt_96_Fe_32_) and eight layers thick. The Pt_3_Fe (200) surface model was chosen with 144 atoms (i.e. Pt_108_Fe_36_) and eight layers thick. The vacuum thickness was set to be 10 Å. The reciprocal space integration was performed using the mesh of 2 × 2 × 1 [55] with Gamma-center-off, which was self-consistently selected for total energy minimization. With these special *k*-points, the total energy converged to less than 5.0 × 10^−7 ^eV per atom. The Hellmann-Feynman forces on the atom converged to less than 0.001 eV/Å.

The Fe and Pt norm-conserving pseudopotentials were generated using the OPIUM code in the Kleinman-Bylander projector form [56], the non-linear partial core correction [57] and a scalar relativistic averaging scheme [58] are used to treat the spin-orbital coupling effect. For this treatment, we actually similarly chose a non-linear core correction technique for correcting the valence-core charge density overlapping in such heavy fermions elements, the details of this method are given in a previous work [47,48]. In particular, we treated the (3*d,* 4*s*, 4*p*) and (5*d*, 6*s*, 6*p*) states as the valence states of both Fe and Pt atoms. The RRKJ method was chosen for optimization of the pseudopotentials [59].

For all of the electronic state calculations, we used self-consistent determination for the U correction on the localized d orbitals to correct the on-site Coulomb energy of the electron spurious self-energy. By that method, the Hubbard U parameters on the half-filled shell of 3d^6^ orbitals of Fe are self-consistently determined to be U_d_ = 6.03 eV, and U_d_ = 5.26 eV for Pt-5d^9^ within the Pt_3_Fe system.

## Supplementary Material

nwaa088_Supplemental_File

## References

[1] Chen JG , Crooks RM, Schrock RR et al. Beyond fossil fuel-driven nitrogen transformations. Science 2018; 360: eaar6611. 29798857 10.1126/science.aar6611 PMC6088796

[2] Jia H , Du A, Zhang CY et al. Site-selective growth of crystalline ceria with oxygen vacancies on gold nanocrystals for near-infrared nitrogen photofixation. J Am Chem Soc 2019; 141: 5083–6. 30897901 10.1021/jacs.8b13062

[3] Liu S , Wang M, Yan CL et al. Facilitating nitrogen accessibility to boron-rich covalent organic frameworks via electrochemical excitation for efficient nitrogen fixation. Nat Commun 2019; 10: 3898. 31467283 10.1038/s41467-019-11846-x PMC6715660

[4] Li L , Tang C, Qiao SZ et al. Two-dimensional mosaic bismuth nanosheets for highly selective ambient electrocatalytic nitrogen reduction. ACS Catal 2019; 9: 2902–8.

[5] Yu X , Han P, Zheng GF et al. Boron-doped graphene for electrocatalytic N_2_ reduction. Joule 2018; 2: 1610–22.

[6] Guo C , Ran J, Qiao SZ et al. Rational design of electrocatalysts and photo(electro)catalysts for nitrogen reduction to ammonia (NH_3_) under ambient conditions. Energy Environ Sci 2018; 11: 45–56.

[7] Hu C , Chen X, Xiong YJ et al. Surface plasmon enabling nitrogen fixation in pure water through a dissociative mechanism under mild conditions. J Am Chem Soc 2019; 141: 7807–14. 31038309 10.1021/jacs.9b01375

[8] Qiu W , Xie X, Sun XP et al. High-performance artificial nitrogen fixation at ambient conditions using a metal-free electrocatalyst. Nat Commun 2018; 9: 3485. 30154483 10.1038/s41467-018-05758-5 PMC6113289

[9] Zhang N , Jalil A, Xiong YJ et al. Refining defect states in W_18_O_49_ by Mo doping: a strategy for tuning N_2_ activation towards solar-driven nitrogen fixation. J Am Chem Soc 2018; 140: 9434–43. 29975522 10.1021/jacs.8b02076

[10] Yao Y , Zhu S, Shao MH et al. A spectroscopic study on the nitrogen electrochemical reduction reaction on gold and platinum surfaces. J Am Chem Soc 2018; 140: 1496–501. 29320173 10.1021/jacs.7b12101

[11] Tang C , Qiao SZ. How to explore ambient electrocatalytic nitrogen reduction reliably and insightfully. Chem Soc Rev 2019; 48: 3166–80. 31107485 10.1039/c9cs00280d

[12] Cheng H , Ding LX, Chen GF et al. Molybdenum carbide nanodots enable efficient electrocatalytic nitrogen fixation under ambient conditions. Adv Mater 2018; 30: 1803694. 10.1002/adma.201803694 30276883

[13] Cheng H , Cui P, Wang HH et al. High efficiency electrochemical nitrogen fixation achieved with a lower pressure reaction system by changing the chemical equilibrium. Angew Chem Int Ed 2019; 58: 15541–7. 10.1002/anie.201910658 31502747

[14] Luo Y , Chen G-F, Ding L et al. Efficient electrocatalytic N_2_ fixation with MXene under ambient conditions. Joule 2019; 3: 279–89.

[15] Hui L , Xue Y, Li YL et al. Highly efficient and selective generation of ammonia and hydrogen on a graphdiyne-based catalyst. J Am Chem Soc 2019; 141: 10677–83. 31149825 10.1021/jacs.9b03004

[16] Geng Z , Liu Y, Zeng J et al. Achieving a record high yield rate of 120.9 μg h^−^^1^ mg^−^^1^_cat_ for N_2_ electrochemical reduction over Ru single-atom catalysts. Adv Mater 2018; 30: 1803498. 10.1002/adma.201803498 30095855

[17] Hong W , Lu W, Qiang W et al. Ambient electrosynthesis of ammonia: electrode porosity and composition engineering. Angew Chem Int Ed 2018; 57: 12360–4. 10.1002/anie.201805514 29923667

[18] Zhang L , Ji X, Sun XP et al. Electrochemical ammonia synthesis via nitrogen reduction reaction on a MoS_2_ catalyst: theoretical and experimental studies. Adv Mater 2018; 30: 1800191. 10.1002/adma.201800191 29808517

[19] Tao H , Choi C, Sun Z et al. Nitrogen fixation by Ru single-atom electrocatalytic reduction. Chem 2018; 5: 204–14.

[20] Wang M , Liu S, Yan CL et al. Over 56.55% Faradaic efficiency of ambient ammonia synthesis enabled by positively shifting the reaction potential. Nat Commun 2019; 10: 341. 30664636 10.1038/s41467-018-08120-x PMC6341113

[21] Shi M , Bao D, Jiang Q et al. Anchoring PdCu amorphous nanocluster on graphene for electrochemical reduction of N_2_ to NH_3_ under ambient conditions in aqueous solution. Adv Energy Mater 2018; 8: 1800124.

[22] Wang H , Li Y, Wang L et al. One-pot synthesis of bi-metallic PdRu tripods as an efficient catalyst for electrocatalytic nitrogen reduction to ammonia. J Mater Chem A 2019; 7: 801–5.

[23] Shi M , Bao D, Jiang Q et al. Au sub-nanoclusters on TiO_2_ toward highly efficient and selective electrocatalyst for N_2_ conversion to NH_3_ at ambient conditions. Adv Mater 2017; 29: 1606550. 10.1002/adma.201606550 28240391

[24] Bao D , Zhang Q, Zhang XB et al. Electrochemical reduction of N_2_ under ambient conditions for artificial N_2_ fixation and renewable energy storage using N_2_/NH_3_ cycle. Adv Mater 2017; 29: 1604799. 10.1002/adma.201604799 27859722

[25] Chen G , Cao X, Wang H et al. Ammonia electrosynthesis with high selectivity under ambient conditions via a Li^+^ incorporation strategy. J Am Chem Soc 2017; 139: 9771–4. 28693318 10.1021/jacs.7b04393

[26] Lv C , Qian Y, Yu GH et al. Defect engineering metal-free polymeric carbon nitride electrocatalyst for effective nitrogen fixation under ambient conditions. Angew Chem Int Ed 2018; 57: 10246–50. 10.1002/anie.201806386 29947048

[27] Lv C , Yan C, Yu GH et al. An amorphous noble-metal-free electrocatalyst enables N_2_ fixation under ambient conditions. Angew Chem Int Ed 2018; 130: 6181–4. 10.1002/anie.201801538 29473991

[28] Yao Y , Wang H, Shao MH et al. Electrochemical nitrogen reduction reaction on ruthenium. ACS Energy Lett. 2019; 4: 1336–41.

[29] Skúlason E , Bligaard T, Nørskov JK et al. A theoretical evaluation of possible transition metal electro-catalysts for N_2_ reduction. Phys Chem Phys 2012; 14: 1235–45. 10.1039/c1cp22271f 22146855

[30] Liu X , Jiao Y, Qiao SZ et al. Building up a picture of the electrocatalytic nitrogen reduction activity of transition metal single-atom catalysts. J Am Chem Soc 2019; 141: 9664–72. 31145607 10.1021/jacs.9b03811

[31] Bai S , Bu L, Huang XQ et al. Multicomponent Pt-based zigzag nanowires as selectivity controllers for selective hydrogenation reactions. J Am Chem Soc 2018; 140: 8384–7. 29924607 10.1021/jacs.8b03862

[32] Luo M , Sun Y, Guo SJ et al. Stable high-index faceted Pt skin on zigzag-like PtFe nanowires enhances oxygen reduction catalysis. Adv Mater 2018; 30: 1705515. 10.1002/adma.201705515 29333666

[33] Tian N , Zhou ZY, Wang ZL et al. Synthesis of tetrahexahedral platinum nanocrystals with high-index facets and high electro-oxidation activity. Science 2007; 316: 732–5. 17478717 10.1126/science.1140484

[34] Zhang L , Zhang JW, Zheng LS et al. Cu^2+^-assisted synthesis of hexoctahedral Au-Pd alloy nanocrystals with high-index facets. J Am Chem Soc 2011; 133: 17114–7. 21894987 10.1021/ja2063617

[35] Bu LZ , Shao Q, Huang XQ et al. PtPb/PtNi intermetallic core/atomic layer shell octahedra for efficient oxygen reduction electrocatalysis. J Am Chem Soc 2017; 139: 9576–82. 28657302 10.1021/jacs.7b03510

[36] Wang Y , Cui X, Zheng W et al. Rational design of Fe-N/C hybrid for enhanced nitrogen reduction electrocatalysis under ambient conditions in aqueous solution. ACS Catal 2018; 9: 336–44.

[37] Wang J , Yu L, Feng X et al. Ambient ammonia synthesis via palladium-catalyzed electro-hydrogenation of dinitrogen at low overpotential. Nat Commun 2018; 9: 1795. 29765053 10.1038/s41467-018-04213-9 PMC5953946

[38] Zhang L , Ding LX, Wang H et al. Ammonia synthesis under ambient conditions: selective electroreduction of dinitrogen to ammonia on black phosphorus nanosheets. Angew Chem Int Ed 2019; 131: 2638–42. 10.1002/anie.201813174 30560583

[39] Liu Y , Su Y, Zhao J et al. Facile ammonia synthesis from electrocatalytic N_2_ reduction under ambient conditions on N-doped porous carbon. ACS Catal 2018; 8: 1186–91.

[40] Zhang X , Luo Z, Zhang H et al. Lithiation-induced amorphization of Pd_3_P_2_S_8_ for highly efficient hydrogen evolution. Nat Catal 2018; 1: 460–8.

[41] Lai J , Huang B, Guo SJ et al. Strongly coupled nickel–cobalt nitrides/carbon hybrid nanocages with Pt-like activity for hydrogen evolution catalysis. Adv Mater 2019; 31: 1805541. 10.1002/adma.201805541 30417441

[42] Kim D , Resasco J, Yang PD et al. Synergistic geometric and electronic effects for electrochemical reduction of carbon dioxide using gold-copper bimetallic nanoparticles. Nat Commun 2014; 5: 4948. 25208828 10.1038/ncomms5948

[43] Duchesne PN , Li ZY, Zhang P et al. Golden single-atomic-site platinum electrocatalysts. Nat Mater 2018; 17: 1033–9. 30250176 10.1038/s41563-018-0167-5

[44] Huang H , Jia H, Zeng J et al. Understanding of strain effects in the electrochemical reduction of CO_2_: using Pd nanostructures as an ideal platform. Angew Chem Int Ed 2017; 129: 3648–52. 10.1002/anie.201612617 28217911

[45] Clark SJ , Segall MD, Payne MC et al. First principles methods using CASTEP. Zeitschrift Fur Kristallographie 2005; 220: 567.

[46] Vladimir IA , Lichtenstein AI. First-principles calculations of the electronic structure and spectra of strongly correlated systems: the LDA+ *U* method. J Phys Condens Matter 1997; 9: 767–807.

[47] Huang B , Gillen R, Robertson J. Study of CeO_2_ and its native defects by density functional theory with repulsive potential. J Phys Chem C 2014; 118: 24248–56.

[48] Huang B . Superiority of DFT+U with non-linear core correction for open-shell binary rare-earth metal oxides: a case study of native point defects in cerium oxides. Philo Mag 2014; 94: 3052–71.

[49] Huang B . 4f fine-structure levels as the dominant error in the electronic structures of binary lanthanide oxides. J Comput Chem 2016; 37: 825–35. 26666512 10.1002/jcc.24272

[50] Huang B . Intrinsic deep hole trap levels in Cu_2_O with self-consistent repulsive Coulomb energy. Solid State Commun 2016; 230: 49–53.

[51] Huang B . Unraveling energy conversion modeling in the intrinsic persistent upconverted luminescence of solids: a study of native point defects in antiferromagnetic Er_2_O_3_. Phys Chem Phys 2016; 18: 13564–82. 10.1039/c6cp01747a 27140724

[52] Huang B . The screened pseudo-charge repulsive potential in perturbed orbitals for band calculations by DFT+U. Phys Chem Phys 2017; 19: 8008–25. 10.1039/c7cp00025a 28263327

[53] Hu J , Huang B, Yang S et al. Engineering stepped edge surface structures of MoS_2_ sheet stacks to accelerate the hydrogen evolution reaction. Energy Environ Sci 2017; 10: 593–603.

[54] Marzari N , Vanderbilt D, Payne MC. Ensemble density-functional theory for Ab initio molecular dynamics of metals and finite-temperature insulators. Phys Rev Lett 1997; 79: 1337–41.

[55] Probert MIJ , Payne MC. Improving the convergence of defect calculations in supercells: an *ab initio* study of the neutral silicon vacancy. Phys Rev B 2003; 67: 075204–15.

[56] Kleinman L , Bylander DM. Efficacious form for model pseudopotentials. Phys Rev Lett 1982; 48: 1425–8.

[57] Louie SG , Cohen ML. Nonlinear ionic pseudopotentials in spin-density-functional calculations. Phys Rev B 1982; 26: 1738–42.

[58] Grinberg I , Rappe AM. Transferable relativistic Dirac-Slater pseudopotentials. Phys Rev B 2000; 62: 2311–5.

[59] Rappe AM , Joannopoulos JD. Optimized pseudopotentials. Phys Rev B 1990; 41: 1227–31. 10.1103/physrevb.41.1227 9993827

